# Inverse Association between Exercising Blood Pressure Response and Left Ventricular Chamber Size and Mass in Women Who Habitually Resistance Train

**DOI:** 10.3390/healthcare12030353

**Published:** 2024-01-30

**Authors:** Evan L. Matthews, John J. Guers, Meghan G. Ramick, Peter A. Hosick

**Affiliations:** 1 Department of Exercise Science and Physical Education, Montclair State University, Montclair, NJ 07043, USA; hosickp@montclair.edu; 2 Department of Biology, Behavioral Neuroscience and Health Sciences, Rider University, Lawrenceville, NJ 08648, USA; jguers@rider.edu; 3 Department of Kinesiology, West Chester University, West Chester, PA 19383, USA; mramick@wcupa.edu

**Keywords:** left ventricular mass, resistance exercise, systolic blood pressure, women

## Abstract

Exercise is a major modifiable lifestyle factor that leads to temporarily increased systolic blood pressure (SBP), which is thought to influence left ventricular mass normalized to body surface area (LVM/BSA). This relationship has never been studied in women who habitually perform resistance exercise. Purpose: To determine if a direct correlation exists between the SBP response to resistance exercise (change from rest; eSBP) and LVM/BSA in young healthy women who habitually resistance train. Methods: Leg extension resistance exercise was performed while continuously monitoring blood pressure using finger plethysmography. LVM was estimated using echocardiography. Data are shown as mean ± SD. Results: Thirty-one women participated (age 23 ± 3 years, height 164 ± 7 cm, body mass 63.7 ± 10.3 kg). Resting SBP (110 ± 8 mmHg, r = 0.355, *p* = 0.049) was shown to be directly correlated to LVM/BSA (72.0 ± 28.4 g/m^2^). Conversely, eSBP (30.8 ± 14.6 ∆mmHg, r = −0.437, *p* = 0.014) was inversely related to LVM/BSA. eSBP was not correlated to interventricular septum width (0.88 ± 0.12 cm, r = −0.137, *p* = 0.463) or posterior wall thickness (0.91 ± 0.15 cm, r = −0.084, *p* = 0.654). eSBP was inversely related to left ventricle internal diameter during diastole (LVIDd) (4.25 ± 0.33 cm, r = −0.411, *p* = 0.021). Conclusion: Counter to the hypothesis, these data suggest an inverse association between eSBP during resistance exercise and LVM/BSA in healthy young women who resistance train. This relationship is due to a smaller LVIDd with greater eSBP.

## 1. Introduction

Resting blood pressure (BP) is an independent determinant of left ventricular mass (LVM) [1]. Conversely, LVM is a common biomarker of heart pathology [2,3,4] and a useful predictor of hypertension development [2,5,6]. Several chronic nutritional and socio-environmental factors affect resting BP and therefore LVM. These include chronic excesses in caloric [7] or sodium [8] intake, lack of leisure-time physical activity [9], and low educational attainment and income [10]. Additionally, BP is acutely elevated during activities of daily life like exercise, mental tasks/mental stress, cold exposure, and occupational standing [11,12]. These acute pressor responses contribute to the stress on the heart and have a cumulative effect on LVM. Therefore, LVM is actually an index of BP over time and in response to lifestyle/socio-environmental stressors [2]. The factors affecting BP and LVM vary in their level of personal control, but exercise is likely one of the more modifiable factors.

As a modifiable lifestyle factor that influences BP and LVM, exercise is especially appealing because it delivers favorable outcomes on most health parameters and provides the greatest dose–response effect on health at low volumes/intensities [13]. Regular exercise decreases resting BP and body mass in those with elevated levels, which typically leads to favorable decreases in LVM [14]. Importantly, habitual exercise decreases resting systolic BP (SBP) more than resting diastolic BP (DBP) [15], and resting SBP is more closely associated with LVM [1,16]. However, during moderate-to-vigorous intensity exercise, the pressures experienced by the heart display a large acute increase and can independently increase LVM [17,18,19,20] and alter heart function [21,22]. Of note, the exercise-induced increases in LVM are typically considered healthy [21], but may increase linearly with the degree of BP responsiveness during exercise. Thus, it is important to study the BP stresses (e.g., SBP) placed on the heart during exercise as they can be more informative than resting BP alone in those who habitually exercise. Exercising SBP (eSBP) is predictive of hypertension development [23] and is augmented in adults with a genetically increased risk of future hypertension [24]. Therefore, young adults who display a high eSBP may develop greater LVM within subclinical ranges. However, an elevated eSBP also suggests they will eventually develop resting hypertension, which will further increase LVM to approach levels of clinical importance. Interestingly, young adults with a genetically increased risk of future hypertension have higher LVM that is not fully explained by resting SBP [1]. These associations show the importance of answering the following question: does eSBP predict LVM?

Prior research examining the relationship between eSBP and LVM has primarily found a direct association between these variables [12,16,25,26,27,28,29,30,31,32,33,34,35,36,37,38], but this is not a universal finding [12,27,28,30,39,40]. When examining the results that do not support the association between eSBP and LVM, the participants did not habitually exercise [12,27,28,30,39,40]. Studies which have compared habitual exercisers to non-exercise controls have found a significant eSBP-to-LVM relationship only in the habitual exercisers [28,30]. This suggests that exercise promotes greater LVM development in those with greater eSBP only when the exercise stimulus on the heart is routinely applied. Unfortunately, women have often been excluded as research participants [41], and none of the studies which targeted adults who habitually exercise included women. Because of this, the literature on the relationship between eSBP and LVM in women is less clear, with some showing a significant relationship [26,32] and others showing no relationship [12,27]. None of the available literature examines how habitual exercise effects this relationship in women.

With the recent cultural shifts promoting women’s fitness as a part of a normal healthy lifestyle, there has been a surge in popularity of resistance exercise for women [42]. However, none of the prior investigations examining the association between eSBP and LVM examined resistance exercise, regardless of sex. It is possible that this relationship is different when examining resistance exercise as eSBP is greater during resistance exercise compared to aerobic exercise, though it is sustained for less time per session [43] Further, the stresses placed on the heart during resistance exercise are different than during aerobic exercise (i.e., greater afterload but less volume load). This complicates the applicability of prior research findings when assessing the potential relationship between peak eSBP during resistance exercise and LVM in women participating in regular resistance exercise. Importantly, the relationship between muscular fitness (i.e., muscular endurance via 2 min sit-up test) and LVM has been found to be different in men and women with similar military physical training [44]. In that prior study, high muscular endurance in men was associated with greater LVM, while this was not the case in women. Therefore, it is difficult to determine what the relationship will be between eSBP during resistance exercise and LVM in women who habitually participate in resistance exercise.

While exercise is unequivocally good for health, these prior studies suggest individuals with exaggerated eSBP responses may warrant special interventions (e.g., modified exercise programs, dietary, or stress management) to prevent excessive LVM development. Without research examining the relationship between eSBP during resistance exercise in resistance-trained women, we cannot appropriately determine needs or best strategies for potential lifestyle interventions. Based on the literature summarized previously, we hypothesized that a direct correlation exists between eSBP during resistance exercise and LVM in apparently healthy resistance-trained women. 

## 2. Materials and Methods

### 2.1. Participants

The experimental protocols and the process for obtaining informed consent conformed with the Declaration of Helsinki of 1975 and as revised in 2013 and were approved by the Institutional Review Board of Montclair State University. All participants completed the informed consent process prior to the start of data collection. All participants were recruited in and around Montclair State University. Participants were excluded if they had chronic medical conditions likely to affect the results or exercise safety (e.g., diabetes, kidney disease, history of cancer, cardiovascular or pulmonary disease, obesity, current pregnancy, etc.). All participants reported being assigned the female sex at birth (referred to throughout this paper as women) [41,45]. Participants were required to be between the ages of 18 and 35 years old. For this analysis, only participants who reported habitual participation in ≥1 day per week of resistance exercise were included (survey question: “How many days do you perform resistance exercise in a typical week?”). The data for this investigation is part of a larger series of studies. Therefore, the resting echocardiographic and demographic values for 9 of the current participants were also included in a previously published data set [1]. Fifty participants completed the study, but ten were excluded due to poor data quality and nine because they did not resistance train. The remaining sample of 31 were analyzed. Assuming a large effect size (r = 0.5 [46]), a sample size of n = 29 would be required for a bivariate correlation with an alpha = 0.05 and power = 0.80 (G*Power 3.1.9.7). Based on previous research examining aerobically trained men performing aerobic exercise for the association between eSBP and LVM/BSA (n = 32, r = 0.58 [28]), a power analysis suggested a required sample size of n = 20. Similarly, the association between resting SBP and LVM/BSA in a mixed sample of men and women from our laboratory (r = 0.606 [1]) suggested a minimal sample size of n = 18. Therefore, the sample size of the current investigation (n = 31) was deemed to be more than adequate to answer the primary questions posed. 

### 2.2. Screening Visit

Following the informed consent process, participants were asked to fill out questionnaires including questions about their medical history and habitual exercise patterns. Once study eligibility was determined, those eligible participated in a 1 repetition maximum (1RM) protocol using leg extension resistance exercise on a weight stack leg extension machine (XMark Fitness, Shreveport, LA, USA). The machine back support was adjusted so the popliteal and lower back were touching the support pads. Participants were strapped to the machine around the thighs and waist with adjustable Velcro straps. They were permitted to hold the machine handle with their left hand, but not their right hand, to mimic the exercise protocol performed during the exercise visit where the right hand was used for continuous blood pressure monitoring. The 1RM testing protocol was performed in accordance with the National Strength and Conditioning Association recommendations [47]. Briefly, participants performed warm-up sets of 10 repetitions at a subjective weight load that would not require intense effort. Following a ≥1 min rest period, another warm-up set of 5 repetitions with a ~20% increase in weight was performed. Following another ≥1 min rest period, participants began single repetition attempts to achieve their 1RM. Each attempt increased the resistance by 10–20% and was followed by a ≥1 min rest period. The greatest weight lifted for a full leg extension was recorded as the 1RM value. 

### 2.3. Echocardiograph and Exercise Visit

Participants were asked to fast (water allowed) for 4 h, abstain from caffeine for 12 h, and avoid alcohol, over-the-counter drugs, and exercise 24 h prior to the echocardiogram/exercise visit. At the beginning of this visit, height was measured using a standard stadiometer, and body weight and body fat percentage were measured with a bioelectrical impedance device (MC-780U, Tanita, Tokyo, Japan). Height and weight were used to calculate body surface area (BSA); BSA = 0.007184 × weight_kg_^0.425^ × height_cm_^0.725^ [48]. The echocardiographic values for LVM were normalized to BSA (LVM/BSA) for the analysis performed in this manuscript to avoid confounds of body size.

Echocardiographic assessments were made prior to exercise while participants rested in a private exam space. Participants were placed in the left lateral decubitus position. All measurements were made using a GE Vivid i Ultrasound (GE healthcare, Nanjing, China) with a 2–5 MHz cardiac transducer and were performed by a single researcher. Participants underwent an echocardiogram using the parasternal long axis view to assess LVM according to the American Society of Echocardiography [49,50]. As previously reported by our laboratory [1], the parasternal long axis view was obtained with the probe placed near the 3rd intercostal space adjacent to the sternum. Linear measurements of wall thicknesses and ventricular chamber diameter were measured using two-dimensional brightness mode ultrasound and were assessed just below the level of the mitral valve leaflet tips perpendicular to the LV long axis. End-diastole was defined as the first video frame after the mitral valve closed. End-systole was defined as the last frame before the mitral valve opened. LVM was calculated at end-diastole as LVM = 0.8 × 1.04 × [(IVSd + LVIDd + PWTd)^3^ − LVIDd^3^] + 0.6, where IVSd is interventricular septum during diastole, PWTd is posterior wall thickness during diastole, and LVIDd is left ventricular internal diameter during diastole [50]. Relative wall thickness (RWT) was calculated as RWT = (2 × PWTd)/LVIDd [50]. RWT and LVM/BSA were examined together to determine left ventricular geometry category (i.e., normal geometry, concentric remodeling, concentric hypertrophy, or eccentric hypertrophy) per published recommendations [50]. Fractional shortening (FS) was calculated as FS = 100 × ((LVIDd − LVIDs)/LVIDd), where LVIDs is left ventricular internal diameter during systole [50]. Apical views were obtained with the probe placed near the 5th or 6th intercostal space on the body’s side. Left ventricular end-diastole volume (EDV) and end-systole volume (ESV) were assessed using the modified Simpson’s method of biplane disk summation [50]. Biplane stroke volume (SV) was calculated as SV = EDV − ESV. Biplane ejection fraction was calculated as Ejection Fraction = ((EDV − ESV)/EDV) × 100.

Following the echocardiogram, participants were escorted to the Exercise Science Lab at Montclair State University for the exercise protocol. Participants were set up on the leg extension machine as described in the 1RM protocol above. Throughout the exercise protocol, SBP, DBP, heart rate (HR), SV, and total peripheral resistance (TPR) were recorded continuously using a non-invasive blood pressure (NIBP) finger cuff (i.e., finger plethysmography) (ADInstruments, Colorado Springs, CO, USA) attached to a finger of the right hand of the participant according to manufacturer instructions. The blood pressure values obtained using this technique have been well validated [51]. SV and TPR values were derived from the finger pulse tracing using model flow estimation [52]. The right arm of the participant was placed into a sling with the elbow positioned at approximately a 90-degree angle to reduce movement of the recording finger and to maintain its position relative to the heart. LabChart 8 Pro software (ADInstruments, Colorado Springs, CO, USA) was used to record and analyze the data. Resting blood pressure and heart rate were assessed using an automated brachial artery blood pressure cuff on the left arm (Omron, Kyoto, Japan). These measurements were taken during a 5 min seated baseline period prior to the exercise protocol. Participants performed 5 sets of 10 repetitions at 70% 1RM with 1 min rest intervals between leg extension sets. The intensity and number of repetitions were chosen because they closely align with recommendations to improve strength in most adults [53,54]. Rating of perceived exertion (RPE) was recorded using a 6–20 Borg scale immediately following each set of exercise. 

### 2.4. Statistical Analysis

All statistical tests were performed using JASP version 0.18.10 (University of Amsterdam, Amsterdam, The Netherlands). Continuous exercising data (HR, SBP, DBP, SV, and TPR) from LabChart 8 Pro software were analyzed as averages of the 5 min baseline period before exercise and the final 10 s of each set. Post hoc level corrections were applied to finger pressures, so the baseline finger cuff pressures were matched to the baseline brachial artery pressures assessed prior to starting exercise, as performed previously [24]. Change scores were then calculated for all continuously recorded variables as the baseline value subtracted from the data timepoint. A select number of bivariate correlations were also provided using eSBP expressed as a percent change (%∆) from rest ([{Set 5 SBP − Resting SBP}/Resting SBP] × 100). A one-way ANOVA was performed to analyze the eSBP across sets. A Bonferroni post hoc correction was applied to compare set-by-set differences. Peak eSBP (set 5) was used for all analyses that did not compare between sets. Bivariate associations were assessed using Pearson’s correlations. The correlation coefficient (r) was interpreted as 0 (no association), ±0.1 to 0.3 (small), ±0.3 to 0.5 (medium), and ±0.5 to 1.0 (large) [46]. Multiple regression models were created using the enter method to investigate the independent predictors of effects of LVM/BSA and eSBP from significantly correlated variables and physiologically or mathematically linked variables of interest. Multiple regression model coefficients with significant *p* values were compared using the absolute value of the standardized coefficient to interpret their relative importance in predicting the variable of interest. Alpha was set at *p* < 0.05. Data are reported as mean ± standard deviation.

## 3. Results

### 3.1. Participant and Protocol Characteristics

Data from 31 young and healthy women participants who reported regular resistance exercise training were included and analyzed as a part of this investigation. When asked, “Think about a typical resistance exercise session and finish this sentence. During resistance exercise I…”, participants could respond as follows: “(1) often fail to complete a rep because it is too difficult (n = 3); (2) sometimes fail to complete a rep because it is too difficult (n = 24); never fail to complete a rep because it is too difficult (n = 4); I do not do resistance exercise (n = 0)”. When asked, “Think about a typical aerobic exercise session and finish this sentence. During aerobic exercise I can typically…”, participants could respond as follows: “(1) talk, but with difficulty (n = 7); (2) talk, but cannot sing (n = 13); (3) talk easily, and could sing a little (n = 6); I do not do aerobic exercise (n = 5)”. When asked, “Does your work involve vigorous-intensity activity that causes large increases in breathing or heart rate like [carrying or lifting heavy loads, digging or construction work] for at least 10 min continuously?”, participants could respond as follows: “(1) yes (n = 4); (2) no (n = 27)”. When asked, “Does your work involve moderate-intensity activity that causes small increases in breathing or heart rate such as brisk walking [or carrying light loads] for at least 10 min continuously?”, participants could respond as follows: (1) yes (n = 13); (2) no (n = 18). Of the 31 participants reported, within the last 6 months, 6 reported using nicotine (1 daily user), 27 reported alcohol use (0 daily users), and 29 reported caffeine use (15 daily users).

When examining the left ventricular geometry of the participants, 15 displayed normal geometry, 15 displayed concentric remodeling, 1 displayed concentric hypertrophy, and 0 displayed eccentric hypertrophy. Table 1 displays participant and protocol characteristics and their correlations to the measurements of interest in this study, i.e., LVM/BSA and set 5 eSBP. LVM/BSA displayed a direct medium-effect correlation with resting SBP, but an inverse medium-effect correlation with eSBP and exercising DBP (eDBP). LVM/BSA displayed a direct medium-effect correlation with FS. The eSBP response displayed a large-effect direct correlation with eDBP and a medium-effect direct correlation with eHR. eSBP also displayed a medium-effect direct correlation with 1RM and mathematically linked exercising weight (~70% 1RM). eSBP displayed a medium-effect inverse correlation to exercising SV and a medium-effect direct correlation to exercising TPR. The relationship between the eSBP and LVM determinates (variables used in LVM calculation) was not significant for IVSd or PWTd, but displayed a medium-effect inverse correlation with LVIDd. Interestingly, resting SBP displayed a direct medium-effect correlation with IVSd (r = 0.419, *p* = 0.019) and PWTd (r = 0.468, *p* = 0.008), but no correlation to LVIDd (r = 0.159, *p* = 0.393). When examining eSBP (%∆) rather than (∆), similar correlation results were found: LVM/BSA (r = −0.495, *p* = 0.004), IVSd (r = −0.182, *p* = 0.325), PWTd (r = −0.150, *p* = 0.420), and LVIDd (r = −0.452, *p* = 0.010).

Table 1 displays participant and protocol characteristics. Variable-specific Pearson correlation coefficient (r) and *p* values are provided vs. left ventricular mass indexed to body surface area (LVM/BSA) and systolic blood pressure change from rest to exercise (eSBP) during set 5. Correlation statistics between mathematically dependent variables were not conducted. 

### 3.2. Exercise Systolic Blood Pressure Response and Left Ventricular Mass

**Figure 1:**
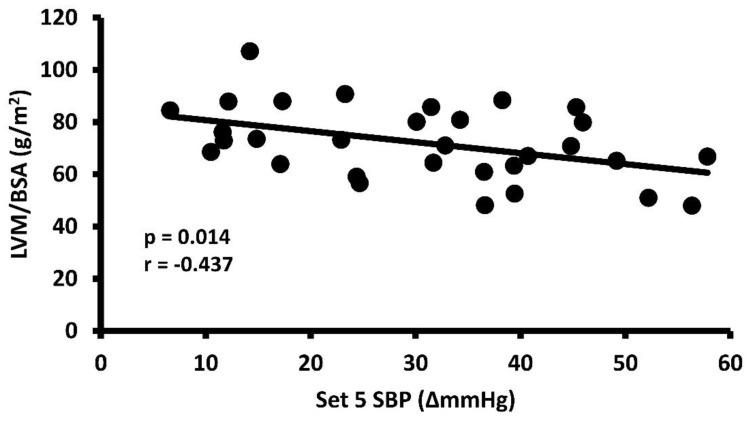
Individual data scatter plot with Pearson correlation coefficient (r) and *p* value for the relationship between peak (set 5) exercising systolic blood pressure response and left ventricular mass/body surface area.

The eSBP response increased with successive sets, reaching peak eSBP during set 5 (ANOVA *p* < 0.001, η^2^ = 0.301) (set 1: 17.8 ± 13.2 ∆mmHg; set 2: 22.6 ± 14.2 ∆mmHg, *p* > 0.05 vs. set 1; set 3: 26.5 ± 12.9 ∆mmHg, *p* < 0.05 vs. set 1, *p* > 0.05 vs. set 2; set 4: 27.1 ± 14.8 ∆mmHg, *p* < 0.05 vs. set 1, *p* > 0.05 vs. sets 2 and 3; set 5: 30.8 ± 14.6 ∆mmHg, *p* < 0.05 vs. sets 1 and 2, *p* > 0.05 vs. sets 3 and 4). Similarly, the inverse relationship between eSBP and LVM/BSA became stronger with successive sets (set 1 r = −0.165, *p* = 0.374; set 2 r = −0.189, *p* = 0.307; set 3 r = −0.374, *p* = 0.038; set 4 r = −0.360, *p* = 0.046; set 5 r = −0.437, *p* = 0.014). The strongest relationship between these variables of interest occurred with peak eSBP during set 5. Individual data points can be seen for the medium-effect inverse relationship between set 5 eSBP and LVM/BSA in Figure 1. 

### 3.3. Independent Effects on Left Ventricular Mass 

A multiple regression model seeking to predict LVM/BSA was conducted using the significant correlates found in Table 1 (i.e., resting SBP and set 5 eSBP) (multiple regression results are found in Table 2). eDBP was excluded from this multiple regression model because its physiological similarity to eSBP led to collinearity. FS was also excluded from this model because they both mathematically depend on LVIDd. The model was statistically significant and both resting SBP and eSBP were independent predictors of LVM/BSA, but in opposite directions. eSBP was a stronger predictor than resting SBP when comparing the standardized coefficients.

### 3.4. Independent Effects on Exercise Systolic Blood Pressure Response

A multiple regression model seeking to predict eSBP was conducted using the significant correlates found in Table 1 (i.e., LVM/BSA and exercising weight) (multiple regression results are found in Table 3). eDBP, eHR, eSV, and eTPR were excluded from this regression model because they are physiologically linked to eSBP and have been included in separate multiple regression models. 1RM was not included in this model because it is mathematically linked to exercising weight. Likewise, LVIDd was not included because it is mathematically linked to LVM and is included in a separate model. This model was statistically significant. LVM/BSA was found to be independent and was a stronger predictor than exercising weight when comparing the standardized coefficients. Exercising weight trended towards, but did not reach, significance within this model (*p* = 0.060).

A multiple regression model seeking to predict eSBP was conducted using the significant correlates found in Table 1 that are methodologically and physiologically linked to eSBP (i.e., eDBP and eHR) (multiple regression results are found in Table 4). This model was statistically significant. eDBP was found to be independent, and was a much stronger predictor than the insignificant eHR when comparing the standardized coefficients. 

A multiple regression model seeking to predict eSBP was conducted using the significant correlates found in Table 1 that are methodologically and physiologically linked to eSBP and use model flow derivation from the shape of the finger pulse signal (i.e., eSV and eTPR) (multiple regression results are found in Table 5). This model trended towards, but did not reach, statistical significance (*p* = 0.056). Neither eSV nor eTPR were found to be independent predictors of eSBP. This collinearity likely results from eTPR being calculated from eSV along with other variables (i.e., heart rate and mean atrial pressure).

Because LVM is a calculated variable, the input variables (i.e., IVSd, LVIDd, PWTd) of that calculation were evaluated within a multiple regression model to determine their individual influence and potential independence in the prediction of eSBP (Table 6). Though the ANOVA for this model was not statistically significant, the coefficient analysis found that LVIDd was an independent inverse predictor of eSBP. Neither IVSd nor PWTd were independent predictors of eSBP. 

## 4. Discussion

In this investigation, we hypothesized a direct correlation between eSBP and LVM/BSA in apparently healthy resistance-trained women. While the two variables appear to be correlated, the relationship was inversed. Both resting SBP and eSBP were independent predictors of LVM/BSA, but in opposite directions. Higher resting SBP was associated with higher LVM/BSA, and higher eSBP was associated with lower LVM/BSA. When attempting to determine the factors that affected eSBP, there were direct correlations with eTPR, maximal muscular strength (i.e., 1RM), and thus the weight lifted during the set (~70% 1RM). This suggests that high muscular strength, and therefore high absolute training load, resulted in greater eTPR and consequently greater eSBP. Others have recently found exercise BP responses to matched relative exercise loads to be affected by muscular strength (i.e., greater absolute exercise loads in those with greater strength) as well [55]. It appears likely that in the present investigation greater training loads resulted in greater compressive forces placed on the blood vessels during resistance exercise movements, thus limiting resistance exercise-mediated vasodilation and decreases in eTPR. It should be noted that the weight lifted during the set was not statically independent (*p* = 0.060) of LVM/BSA as a predictor for eSBP. 

When examining the effect of the variables used to calculate LVM on eSBP, only LVIDd was an independent predictor of eSBP, and it was so with an inverse relationship, suggesting that increased eSBP may lead to a decrease in left ventricular chamber size (i.e., LVIDd). Conversely, resting SBP was not correlated to LVIDd, but was directly correlated to muscle wall thickness (both IVSd and PWTd), leading to a positive association with LVM/BSA. Therefore, our participant population responded differently to SBP during rest and in response to leg extension resistance exercise. Interestingly, women with pathologic aortic stenosis leading to chronic left ventricular pressure overload (experienced at rest and during physical exertion) exhibit exaggerated LVM and shrinking LVIDd compared to males with the same condition [56]. The decreased LVIDd in women with aortic stenosis, and healthy women from the current study with high eSBP is likely an adaptive function to decrease myocardial work [57]. According to Laplace’s law, the tension of a spherical wall is proportional to its radius and pressure yet inversely related to wall thickness.

Wall tension = (transmural pressure × chamber radius)/(2 × wall thickness);

where wall tension ≈ myocardial work, transmural pressure ≈ systolic pressure during the contraction phase of the cardiac cycle, chamber radius ≈ ½ LVIDd, and wall thickness ≈ average of IVSd and PWTd [58].

Thus, a decreased LVIDd would lower myocardial wall tension/work even in the absence of wall thickening. 

In women, low testosterone levels likely decrease the prospect of myocardial muscle thickening. In a 70-day old rat gonadectomy model (i.e., removing endogenous sex hormones) [59], male rats had a decrease in heart mass, which was partially corrected with 3 mg/day of exogenous testosterone administration. In female rats, gonadectomy did not impact heart mass, nor did subsequent supplementation of estrogen, progesterone, or estrogen + progesterone. Subsequent supplementation with 2 mg/day of testosterone did increase heart mass in female gonadectomized rats. These results, and the results of similar animal investigations [60], highlight the important role of testosterone in LVM hypertrophy. In human studies, serum testosterone levels correlate with LVM/BSA in older women [61]. Similarly, exogenous testosterone administration is typically associated with LVM hypertrophy [62], though this is not a universal finding [63]. Estrogens provide a complex and incompletely understood set of direct and indirect effects on the myocardium, but generally play a cardioprotective role [64]. Current understanding suggests that estrogen directly opposes LVM growth due to exercise training using multiple mechanisms [41]. Therefore, the sex hormone profile of women likely leads to a decreased LVIDd in response to high eSBP instead of increased wall thickness. 

Nearly half (n = 15) of the participants in the current investigation displayed concentric remodeling of the left ventricle. This is due to an elevated RWT (normal ≤ 0.42 [50]; study mean = 0.43 (Table 1)), which is due to decreased LVIDd. Despite the lack of increased LVM/BSA in this condition, it is believed that this is an early response to left ventricular pressure overload with a trajectory leading to higher LVM/BSA and concentric hypertrophy [57]. “Physiological hypertrophy” (i.e., healthy hypertrophy) is characterized as an increase in LVIDd and LVM/BSA so that RWT remains normal [57]. This results from equal adaptation to pressure and volume overload and is common in many types of athletes [57]. In the current investigation, only women who resistance train regularly (average 3.7 days/week; Table 1) were included in this analysis, though many also reported regular aerobic training (i.e., primarily volume overload) (average 2.6 days/week; Table 1). An intervention to increase the aerobic training volume of our participants would likely result in the “physiological hypertrophy” phenotype with preserved LVIDd. Importantly, aerobic training is a potent stimulant for SV improvement, which is limited by concentric remodeling of the left ventricle. Therefore, increasing aerobic exercise may be a useful lifestyle intervention in resistance-trained women with elevated resting SBP and eSBP, as our results suggest these individuals would be at the greatest risk for elevated LVM/BSA and reduced LVIDd (i.e., concentric hypertrophy). This intervention may warrant future research.

While the young, healthy participants of the current investigation likely have minimal cardiovascular risk, with aging, the risk of comorbidities like hypertension increases dramatically [65]. This will complicate left ventricular geometry, warranting lifestyle interventions such as aerobic training (discussed above) and dietary interventions to prevent the development of concentric hypertrophy. These may include dietary emphasis on proper weight maintenance [7] and sodium reduction [8]. Other blood pressure-lowering lifestyle interventional strategies may include stress reduction, adequate sleep, and increased leisure-time physical activity [9]. These lifestyle intervention strategies are likely impacted by a variety of socio-environmental factors that lead to accessibility issues and other barriers. 

The current investigation is not without limitations. We studied young women at low risk of current cardiovascular disease. However, cardiovascular disease risk later in life is a result of behaviors across the lifespan. Therefore, it is important to study the early effects of lifestyle choices to better understand how they may impact risk later on. The emphasis of this investigation was on LVM/BSA, which typically becomes dangerous when hypertrophic cardiomyopathy is present. The dogma in the past 30 years for this condition has been that it is a genetic disease rather than a lifestyle disease. However, recent evidence suggests a non-genetic lifestyle component of the disease, as most with the condition test negative for known genetic mutations, and many with these genetic mutations do not develop the condition [66]. Regardless, a high eSBP suggests an increased likelihood of future hypertension. Thus, studying the physiology of young individuals likely to develop pathology later in life is important for characterizing the development of the pathology in its subclinical stages. Additionally, we found that elevated eSBP is associated with decreased LVIDd in young adulthood, which may be complicated later in life when resting blood pressure begins to reach clinically significant levels. Our study sample did not just perform resistance exercise. Therefore, some effects of aerobic exercise likely confound our data. However, there is a strong bias towards resistance training in our sample as the participants completed nearly double the minimum recommended resistance exercise frequency according to the American College of Sports Medicine but did not reach the minimal recommended aerobic exercise frequency. Extensive training histories of the participants were not recorded. Therefore, conclusions regarding training experience (e.g., months or years) or resistance training focus (e.g., muscle power vs. local muscular endurance) cannot be determined. However, resistance training characteristics (e.g., sets, repetitions, movement speed, etc.) usually change over time and vary person-to-person at any one point in time, likely limiting the usefulness of such data in a heterogeneous group. Likewise, we did not collect data on some potentially confounding variables such as socio-economic status, aerobic fitness level, activities of daily living, nutritional intakes, etc. Future studies should determine the modulating impact of these and other confounding variables. The current investigation has a cross-sectional study design, limiting mechanistic insight. However, the mechanisms discussed here are supported by other experimental studies in varying populations and different stress-inducing protocols. Despite this, the authors believe longitudinal testing should be performed to confirm the findings of this investigation. Additionally, cross-sectional studies comparing populations with different sex hormone profiles (e.g., healthy resistance-trained women vs. men) would help to support the proposed mechanisms of this investigation. Therefore, blood biomarker analysis should be conducted in future studies to assess testosterone, estrogen, serum myostatin levels, and other markers of hypertrophy. The current investigation utilized a widely used and validated [51] research technique of non-invasive finger photoplethysmography to continuously monitor blood pressure during the resistance exercise bout. This technique is not perfect, and could have modified our results as it assesses finger blood pressure rather than the standard brachial artery blood pressure. This is important as blood pressure values vary throughout the vascular network [67,68,69]. We attempted to address this by level-correcting the resting finger blood pressure values to resting brachial artery blood pressure values. Finger blood pressure monitoring can also result in errors in blood pressure values when the hand being assessed is not fully relaxed [51]. A single researcher assessed the blood pressure tracings of every participant and excluded participants whose signals included significant error. 

## 5. Conclusions

The current study examined women with habitual resistance training experience and found those with the greatest systolic blood pressure responses had the smallest left ventricular chamber sizes, and therefore the smallest estimated left ventricular chamber masses. Future research should seek to determine if the smaller chamber sizes are a compensatory adaptation of the heart to the high blood pressures experienced during resistance training, as this would decrease wall stress and myocardial work at a given pressure.

## Figures and Tables

**Table 1:** Participant and protocol characteristics relation to main outcome variables.

		LVM/BSA		eSBP	
Variable	Mean ± SD	r	*p*	r	*p*
Age (years)	23 ± 3	0.181	0.330	0.280	0.126
Height (cm)	164 ± 7	-	-	0.072	0.702
Body Mass (kg)	63.7 ± 10.3	-	-	0.151	0.417
BSA (m^2^)	1.69 ± 0.15	-	-	0.117	0.340
Body Fat (%)	25.5 ± 6.5	0.067	0.719	0.258	0.160
Resting SBP (mmHg)	110 ± 8	0.355	0.049	0.074	0.692
Resting DBP (mmHg)	74 ± 8	0.058	0.756	0.016	0.930
Resting HR (beats/min)	73 ± 10	−0.054	0.775	0.039	0.835
eSBP (∆mmHg)	30.8 ± 14.6	−0.437	0.014	-	-
eDBP (∆mmHg)	16.6 ± 8.8	−0.393	0.028	0.736	<0.001
eHR (∆beats/min)	35.3 ± 12.9	−0.178	0.338	0.467	0.008
1RM (kg)	68 ± 24	−0.153	0.412	0.370	0.040
Exercising Weight (kg)	49 ± 17	−0.169	0.364	0.381	0.034
R Ex (Days/week)	3.7 ± 1.6	−0.276	0.132	0.189	0.306
A Ex (Days/week)	2.6 ± 1.9	0.200	0.283	−0.311	0.088
Sedentary Time (min/day)	457 ± 205	−0.016	0.932	−0.209	0.266
MF Resting SV (mL/beat)	36.0 ± 7.1	0.097	0.603	−0.261	0.155
MF eSV (mL/beat)	34.0 ± 7.8	0.312	0.087	−0.426	0.017
MF Resting TPR (mmHg/mL/s)	2.23 ± 0.70	−0.109	0.558	0.234	0.205
MF eTPR (mmHg/mL/s)	1.99 ± 0.68	−0.307	0.092	0.396	0.027
Biplane Resting EDV (mL)	70.6 ± 14.0	0.157	0.408	−0.233	0.214
Biplane Resting ESV (mL)	28.1 ± 7.5	0.094	0.621	−0.218	0.248
Biplane Resting SV (mL/beat)	42.5 ± 9.1	0.165	0.383	−0.183	0.333
Biplane Ejection Fraction (%)	60.3 ± 6.0	0.015	0.938	0.122	0.520
LVM/BSA (g/m^2^)	72.0 ± 28.4	-	-	−0.437	0.014
IVSd (cm)	0.88 ± 0.12	-	-	−0.137	0.463
LVIDd (cm)	4.25 ± 0.33	-	-	−0.411	0.021
LVIDs (cm)	2.86 ± 0.26	0.304	0.095	−0.177	0.340
PWTd (cm)	0.91 ± 0.15	-	-	−0.084	0.654
RWT (Unitless)	0.43 ± 0.08	0.227	0.219	0.103	0.582
FS (%)	32.8 ± 4.5	0.411	0.021	−0.246	0.182

SBP, systolic blood pressure; DBP, diastolic blood pressure; HR, heart rate; eDBP, diastolic blood pressure change from rest to set 5 during exercise; eHR, heart rate change from rest to set 5 during exercise; 1RM, 1 repetition maximum; R Ex, resistance exercise; A Ex, aerobic exercise; MF, model flow-derived value from finger plethysmography; SV, stroke volume; eSV, stroke volume during set 5 of exercise; TPR, total peripheral resistance; eTPR, total peripheral resistance during set 5 of exercise; Biplane, derived from echocardiography using Simpson’s biplane method of disks; IVSd, interventricular septum thickness during diastole; LVIDd, left ventricle internal diameter during diastole; LVIDs, left ventricle internal diameter during systole; PWTd, posterior wall thickness during diastole; RWT, relative wall thickness; FS, fractional shortening.

**Table 2:** Multiple regression for LVM/BSA with significant bivariate coefficients.

ANOVA	R	R^2^	df	*p*
LVM/BSA	0.585	0.342	2	0.003 *
Coefficients	Unstandardized	Standard Error	Standardized	*p*
Intercept	14.391	28.317	-	0.615
Resting SBP (mmHg)	0.650	0.257	0.389	0.017 *
eSBP (∆mmHg)	−0.449	0.148	−0.466	0.005 *

LVM/BSA, left ventricular mass indexed to body surface area; resting SBP, resting systolic blood pressure; eSBP, systolic blood pressure change from rest to during exercise (set 5 used). * Indicates a significant *p* value.

**Table 3:** Multiple regression for eSBP with significant bivariate coefficients.

ANOVA	R	R^2^	df	*p*
eSBP	0.537	0.289	2	0.009 *
Coefficients	Unstandardized	Standard Error	Standardized	*p*
Intercept	46.166	14.987	-	0.005 *
LVM/BSA (g/m^2^)	−0.398	0.168	−0.384	0.025 *
Exercising Weight (kg)	0.271	0.139	0.317	0.060

eSBP, systolic blood pressure change from rest to during exercise (set 5 used); LVM/BSA, left ventricular mass indexed to body surface area. * Indicates a significant *p* value.

**Table 4:** Multiple regression for eSBP with significant physiologically linked coefficients.

ANOVA	R	R^2^	df	*p*
eSBP	0.740	0.548	2	<0.001 *
Coefficients	Unstandardized	Standard Error	Standardized	*p*
Intercept	8.338	5.468	-	0.139
eDBP (∆mmHg)	1.133	0.251	0.686	<0.001 *
eHR (∆beats/min)	0.104	0.172	0.091	0.552

eSBP, systolic blood pressure change from rest to during exercise (set 5 used); eDBP, diastolic blood pressure change from rest to set 5 during exercise; eHR, heart rate change from rest to set 5 during exercise. * Indicates a significant *p* value.

**Table 5:** Multiple regression for eSBP with significant model flow-derived coefficients.

ANOVA	R	R^2^	df	*p*
eSBP	0.432	0.187	2	0.056
Coefficients	Unstandardized	Standard Error	Standardized	*p*
Intercept	45.159	31.745	-	0.166
eSV (mL)	−0.588	0.581	−0.315	0.320
eTPR (mmHg/mL/s)	2.829	6.657	0.132	0.674

eSBP, systolic blood pressure change from rest to during exercise (set 5 used); eSV, stroke volume during set 5 of exercise; eTPR, total peripheral resistance during set 5 of exercise.

**Table 6:** Multiple regression for eSBP with LVM determinates.

ANOVA	R	R^2^	df	*p*
eSBP	0.426	0.181	3	0.139
Coefficients	Unstandardized	Standard Error	Standardized	*p*
Intercept	116.829	36.627	-	0.004 *
IVSd (cm)	7.216	28.135	0.057	0.800
LVIDd (cm)	−18.919	8.178	−0.433	0.029 *
PWTd (cm)	−13.138	21.011	−0.135	0.537

eSBP, systolic blood pressure change from rest to during exercise (set 5 used); IVSd, interventricular septum thickness during diastole; LVIDd, left ventricle internal diameter during diastole; PWTd, posterior wall thickness during diastole. * Indicates a significant *p* value.

## Data Availability

The data presented in this study are openly available in Montclair State University Digital Commons at https://digitalcommons.montclair.edu/data/11 (accessed on 1 January 2024), listed under the manuscript title, “Inverse Association between Exercising Blood Pressure Response and Left Ventricular Chamber Size and Mass in Women Who Habitually Resistance Train”.
